# Association of *Glutathione S transferases* Polymorphisms with Glaucoma: A Meta-Analysis

**DOI:** 10.1371/journal.pone.0054037

**Published:** 2013-01-14

**Authors:** Yibo Yu, Yu Weng, Jing Guo, Guangdi Chen, Ke Yao

**Affiliations:** 1 Eye Center of the 2^nd^ Affiliated Hospital, Zhejiang University School of Medicine, Hangzhou, China; 2 Department of Clinical Laboratory, Sir Run Run Shaw Hospital, Zhejiang University School of Medicine, Hangzhou, China; 3 Department of Public Health, Zhejiang University School of Medicine, Hangzhou, China; Zhongshan Ophthalmic Center, China

## Abstract

**Background:**

*Glutathione S transferase* (*GST*) polymorphisms have been considered risk factors for the development of glaucoma, including primary open angle glaucoma (POAG) and other types of glaucoma. However, the results remain controversial. In this study, we have conducted a meta-analysis to assess the association between polymorphisms of *GSTM1*, *GSTT1* and *GSTP1* and glaucoma risk.

**Methods:**

Published literature from PubMed and other databases were retrieved. All studies evaluating the association between *GSTM1*, *GSTT1* and *GSTP1* polymorphisms and glaucoma risk were included. Pooled odds ratio (OR) and 95% confidence interval (CI) were calculated using random- or fixed-effects model.

**Results:**

Twelve studies on *GSTM1* (1109 cases and 844 controls), ten studies on *GSTT1* (709 cases and 664 controls) and four studies on *GSTP1* (543 cases and 511 controls) were included. By pooling all the studies, either *GSTM1* or *GSTT1* null polymorphism was not associated with a POAG risk, and this negative association maintained in Caucasian. The *GSTP1* Ile 105 Val polymorphism was significantly correlated with increased POAG risk among Caucasian in a recessive model (Val/Val *vs*. Ile/Ile+Ile/Val: OR, 1.62, 95%CI: 1.00–2.61). Interestingly, increased glaucoma risk was associated with the combined *GSTM1* and *GSTT1* null genotypes (OR, 2.20; 95% CI, 1.47–3.31), and with the combined *GSTM1* null and *GSTP1* Val genotypes (OR, 1.86; 95% CI, 1.15–3.01).

**Conclusions:**

This meta-analysis suggests that combinations of *GST* polymorphisms are associated with glaucoma risk. Given the limited sample size, the associations between single *GST* polymorphism and glaucoma risk await further investigation.

## Introduction

Glaucoma is a heterogeneous group of diseases characterized by the death of the retinal ganglion cells and progressive degeneration of the optic nerve. It is the second most frequent cause of irreversible blindness in the world and affects primarily the older population, estimated to affect about 80 million people worldwide by 2020 [1]. However, the etiology of glaucoma remains obscure. Risk factors for glaucoma include aging, elevated intraocular pressure, variable susceptibility of the optic nerve, vascular factors (ischemia), diabetes, myopia, cigarette smoking and positive family history [2]. Glaucoma can be inherited as a Mendelian autosomal-dominant or autosomal-recessive trait, or as a complex multifactorial trait [3]. Genetic approaches have defined the causative genes (e.g., *MYOC*, *OPTN* and *WDR36*) for juvenile-onset and late-onset primary open angle glaucoma (POAG) [4]. In addition to these genes, over 20 gene variants were found to be associated with glaucoma [5]. Recently, large-scale genome-wide association studies have been conducted to map the genes for glaucoma [6], [7], [8].

Growing evidence supports the involvement of oxidative stress as a common component of glaucomatous neurodegeneration in different subcellular compartments of retinal ganglion cells (RGCs), by acting as a second messenger and/or modulating protein function by redox modifications of downstream effectors through enzymatic oxidation of specific substrates [9]. There are many defensive mechanisms against this oxidative damage, including catalase, superoxide dismutase, glutathione peroxidase, and glutathione S transferase (GST) in the eye for protection. Among them, GST is a multigene family with different enzymes that play an important role in the anti-oxidation, detoxification and elimination of xenobiotics, including carcinogens, oxidants, toxins, and drugs [10]. Human GST enzymes mainly include members of eight classes, assigned on the basis of sequence similarity: Alpha (GSTA), Mu (GSTM), Pi (GSTP), Theta (GSTT), Kappa (GSTK), Zeta (GSTZ), Omega (GSTO), and Sigma (GSTS) [11].

Previous studies of allelic variants in these classes have identified two major polymorphisms of the *GSTT1* and *GSTM1* genes caused by a deletion in each gene, and a single-nucleotide polymorphism (SNP) of *GSTP1* resulting in the coding sequence change Ile 105 Val [11]. The deletion of *GSTT1* or *GSTM1*, or *GSTP1* Ile 105 Val polymorphism results in an absence of their enzyme activity [12], [13], [14], and these polymorphisms of *GST* have been associated with altered risk of a variety of pathologies including cancer [15], cardiovascular disease [16], respiratory disease [17], and ophthalmologic problems such as cataract [18], [19]. The relationship between *GST* polymorphisms and risk of glaucoma has been studied for more than 10 years. Several studies have found *GST* polymorphisms to be protective or risk factors in POAG [20], [21], [22], [23], [24], [25], [26] or other types of glaucoma [27], but other studies show no association between *GST* polymorphisms and risk of glaucoma [28], [29]. These studies revealed an inconsistent conclusion, probably due to the relatively small size of subjects, since individual studies are usually underpowered in detecting the effect of low penetrance genes; therefore, in this study we conducted a meta-analysis to investigate the associations between *GSTM1*, *GSTT1*, and *GSTP1* polymorphisms and the risk for glaucoma.

## Materials and Methods

### Identification and Eligibility of Relevant Studies

To identify all articles that examined the association of *GST* polymorphism with glaucoma, we conducted a literature search in the PubMed databases up to August 2012 using the following MeSH terms and keywords: “glutathione S transferase”, “polymorphism” and “glaucoma”. Additional studies were identified by a manual search from other sources (e.g., Web of Knowledge), references of original studies or review articles on this topic. Eligible studies included in this meta-analysis had to meet the following criteria: (a) evaluation of the association between *GSTM1* or *GSTT1* null genotypes, or *GSTP1* Ile 105 Val polymorphism and glaucoma, (b) an unrelated case-control study, if studies had partly overlapped subjects, only the one with a larger sample size was selected, (c) available genotype frequency, (d) sufficient published data for estimating an odds ratio (OR) with 95% confidence interval (CI), and (e) papers published in English from 2000.

### Data Extraction

Two investigators independently assessed the articles for inclusion/exclusion and extracted data, and reached a consensus on all of the items. For each study, the following information was extracted: name of the first author; publication year; ethnicity (country); sample size (numbers of cases and controls); gene polymorphisms investigated; types of glaucoma; sources of samples; genotyping methods.

### Statistical Analysis

The association between *GSTM1*, *GSTT1* or *GSTP1* polymorphism and glaucoma was estimated by calculating pooled odd ratios (ORs) and 95% CIs. The significance of the pooled OR was determined by Z test (*P*<0.05 was considered statistically significant). The risk of *GSTM1* or *GSTT1* null genotype on glaucoma was evaluated by comparing with their reference wild type homozygote. For the *GSTP1* polymorphism, we first estimated the risks of the Ile/Val and Val/Val genotypes on glaucoma, compared with the reference Ile/Ile homozygote, and then evaluated the risks of (Ile/Val+Val/Val *vs*. Ile/Ile) and (Val/Val *vs*. Ile/Ile+Ile/Val) on glaucoma, assuming dominant and recessive effects of the variant Val/Val allele, respectively. The I^2^-based Q statistic test was performed to evaluate variations due to heterogeneity rather than chance. A random-effects (DerSimonian-Laird method) or fixed-effects (Mantel-Haenszel method) model was used to calculate pooled effect estimates in the presence (*P*≤0.10) or absence (*P*>0.10) of heterogeneity. Publication bias was detected by Egger’s test [30] and Begg’s [31] test for the overall pooled analysis of *GSTM1* and *GSTT1* null genotypes, and recessive model of *GSTP1*. Additionally, Begg’s funnel plot was drawn. Asymmetry of the funnel plot means a potential publication bias. Stratified analyses were also performed by types of glaucoma and ethnicities of study populations. For the one-way sensitivity analysis, one single study was excluded each time, and the new pooled results could reflect the influence of that deleted study to the overall summary OR. All analyses were done with Stata software (version 11.0; Stata Corp LP, College Station, TX), using two-sided *P* values.

## Results

### Characteristics of Studies

**Figure 1:**
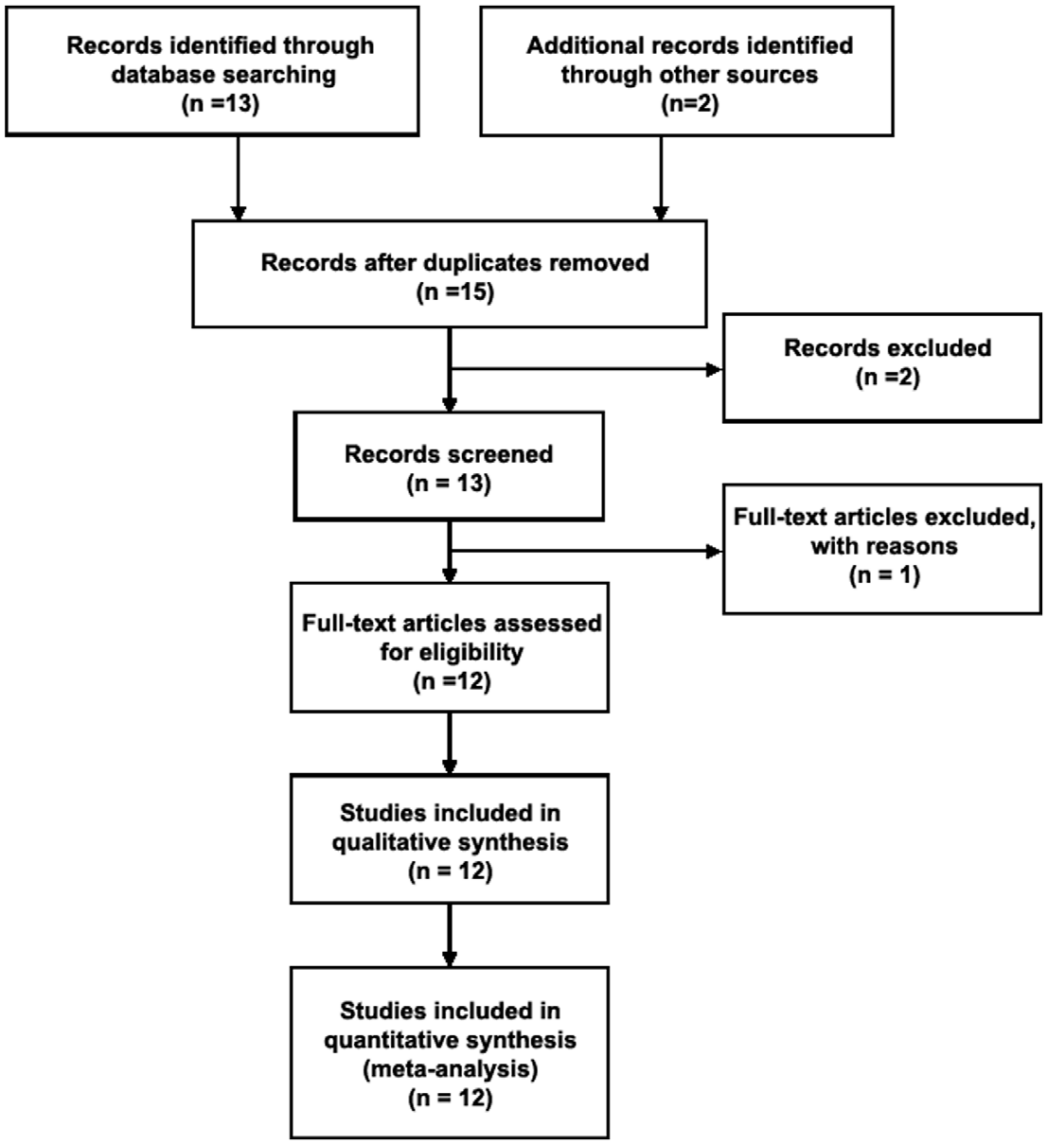
Flow diagram of studies identification.

Thirteen abstracts were retrieved through the search “glutathione S transferase”, “polymorphism” and “glaucoma”, and ten studies meeting the inclusion criteria were identified as eligible [20], [22], [23], [24], [26], [27], [28], [29], [32], [33]. Out of the thirteen, one was commentary [34], and one was *in vitro* study to evaluate the sensitivity to oxidative stress of anterior chamber tissues [35]. One article was excluded due to the study on the relationship between GST polymorphisms and risk of age-related macular degeneration [36]. We also included two eligible studies with manual searching [21], [25]. As a result, a total of twelve studies met the inclusion criteria and were identified as eligible articles (Figure 1).

Twelve studies were included in the meta-analysis of *GSTM1* genotype (1908 cases, 1457 controls), ten studies for *GSTT1* genotype (1414 cases, 1177 controls) and four studies for *GSTP1* polymorphism (543 cases, 511 controls). For the ethnicities, eleven studies of Caucasians and one study of Asians were included on the *GSTM1* genotype. As to *GSTT1*, night studies of Caucasians and one study of Asians were included. The four studies on *GSTP1* were all based on the Caucasians. For the glaucoma type, this meta-analysis included ten, eight, and three studies on the relationship between *GSTM1*, *GSTT1*, and *GSTP1* polymorphism and risk of POAG (the most common form of glaucoma), respectively. In addition, we included four and three studies on the association between the *GSTM1* and *GSTT1* polymorphism and risk of other types of glaucoma (including exfoliative glaucoma and primary closed angle glaucoma), respectively. In addition to the study by Juronen et al. [20], in which the *GSTM1* and *GSTT1* phenotypes were determined with monoclonal antibody based enzyme-linked immunosorbent assay (ELISA), the genotyping for *GSTM1*, *GSTT1* or GSTP1 was performed using polymerase chain reaction (PCR) in all other studies. The detailed characteristics of each study included in the meta-analysis are presented in Table 1, and the *GST* polymorphism genotype distributions from each study are presented in Table S1 and S2.

**Table 1:** Characteristics of literatures included in the meta-analysis.

Author/Year [Reference]	Origin	Ethnicity	Case/control	GST family	Glaucoma Type*	Samples	Genotype
Juronen 2000 [20]	Estonia	Caucasian	250/202	GSTM1/GSTT1/GSTP1	POAG	Blood	ELISA
Izzotti 2003 [21]	Italy	Caucasian	45/46	GSTM1/GSTT1	POAG	Trabecular meshwork	PCR
Jansson 2003 [33]	Sweden	Caucasian	388/200	GSTM1	POAG/Others	Blood	PCR
Yilmaz 2005 [28]	Turkey	Caucasian	53/65	GSTM1/GSTT1/GSTP1	Others	Blood	PCR
Yildirim 2005 [22]	Turkey	Caucasian	153/159	GSTM1/GSTT1/GSTP1	POAG	Blood	PCR
Unal 2007 [23]	Turkey	Caucasian	144/121	GSTM1/GSTT1	POAG	Blood	PCR
Abu-Amero 2008 [24]	Saudi Arabia	Caucasian	107/120	GSTM1/GSTT1	POAG/Others	Blood	PCR
Rasool 2010 [25]	Egypt	Caucasian	32/16	GSTM1/GSTT1	POAG	Trabeculectomy specimens	PCR
Fan 2010 [29]	China	Asian	405/201	GSTM1/GSTT1	POAG	Blood	PCR
Khan 2010 [27]	Pakistan	Caucasian	165/162	GSTM1/GSTT1	Others	Blood	PCR
Izzotti 2010 [32]	Italy	Caucasian	100/100	GSTM1	POAG	Trabecular meshwork	PCR
Rocha 2011 [26]	Brazil	Caucasian	87/85	GSTM1/GSTT1/GSTP1	POAG	Blood	PCR

* Others: including exfoliative and primary closed angle glaucoma.

Abbreviations: POAG, primary closed angle glaucoma; PCR, Polymerase chain reaction; ELISA, enzyme-linked immunosorbent assay.

### Quantitative Synthesis

Table 2 shows the results of the meta-analysis on the association between *GSTM1* or *GSTT1* null polymorphism and risk of glaucoma. By pooling all the studies, either *GSTM1* or *GSTT1* null polymorphism was not associated with a glaucoma risk, and this negative association maintained in Caucasian (Table 2, and Figure S1 and S2). When stratified by glaucoma types, no association was found between *GSTM1* or *GSTT1* null polymorphism and risk of POAG, or other types of glaucoma, in all populations or in Caucasians.

**Table 2:** Subgroup Analysis of the Association between *GSTM1* and *GSTT1* Polymorphisms and the Risk for Glaucoma.

Groups	n†	Statistical Method	OR (95% CI)	*P*
**All Glaucoma**				
*GSTM1* null				
Pooled	12	Random	1.25 (0.82- 1.90)	0.290
Caucasian	11	Random	1.25 (0.77- 2.04)	0.361
Asian	1			
*GSTT1* null				
Pooled	10	Random	1.37 (0.82- 2.28)	0.229
Caucasian	9	Random	1.49 (0.83- 2.67)	0.183
Asian	1	1		
**POAG**				
*GSTM1* null				
Pooled	10	Random	1.23 (0.74- 2.03)	0.426
Caucasian	9	Random	1.24 (0.68- 2.27)	0.474
Asian	1			
*GSTT1* null				
Pooled	8	Random	1.42 (0.80- 2.52)	0.237
Caucasian	7	Random	1.61 (0.80- 3.24)	0.182
Asian	1			
**Others** *				
*GSTM1* null				
Pooled	4	Random	1.64 (0.82–3.28)	0.164
*GSTT1* null				
Pooled	3	Random	2.13 (0.59–7.72)	0.248

* Others: including exfoliative and primary closed angle glaucoma.

† n: number of studies.

Abbreviations: POAG, primary closed angle glaucoma.

We also examined the association between *GSTP1* Ile 105 Val polymorphism and glaucoma risk, and the overall result showed that *GSTP1* polymorphism was not correlated with glaucoma risk in all four models by pooling all four studies (Table 3 and Figure S3). In subgroup analysis, we found that *GSTP1* Ile 105 Val polymorphism was significantly correlated with increased POAG risk in a recessive model (Val/Val *vs*. Ile/Ile+ Ile/Val: OR, 1.62; 95%CI, 1.00–2.61; *P* = 0.049) but not in other three models. Interestingly, these three studies were all based on Caucasian populations, thus, *GSTP1* Ile 105 Val polymorphism in recessive model was associated with increased POAG risk in Caucasians (Table 3).

**Table 3:** Meta-analysis of the *GSTP1* Ile105Val polymorphism on glaucoma risk.

Groups	n†	Ile/Val vs. Ile/Ile		Val/Val vs. Ile/Ile		Ile/Val +Val/Val vs. Ile/Ile (dominant)		Val/Val vs. Ile/Ile +Ile/Val (recessive)	
		OR (95% CI)	*P*	OR (95% CI)	*P*	OR (95% CI)	*P*	OR (95% CI)	*P*
All glaucoma									
Pooled	4	0.95(0.73–1.24)	0.700	1.17(0.51–2.68)	0.706‡	1.00(0.78–1.29)	0.987	1.18(0.50–2.80)	0.708‡
Glaucoma type									
POAG	3	0.92(0.70–1.23)	0.587	1.55(0.94–2.55)	0.087	1.03(0.79–1.34)	0.836	1.62(1.00–2.61)	0.049
Others*	1								

* Others: including exfoliative glaucoma.

† Number of studies.

‡ A random-effects model was used to calculate pooled effect estimates.

Abbreviations: POAG, primary closed angle glaucoma.

To investigate if the profiles of *GST* genotypes were associated with the risk of glaucoma, we first examined the association between combinations of *GSTM1* and *GSTT1* null genotypes and the risk of glaucoma, in which the reference group consisted of individuals with both putative low-risk genotypes, i.e., the presence of *GSTM1* and *GSTT1* genotypes [22]. Table 4 displays the risk of glaucoma associated with combinations of *GST* null genotypes as well as the trend in risk associated with each putative high-risk null genotype. The data showed a significant association between increased glaucoma risk and the combined *GSTM1* and *GSTT1* null genotypes in all population (OR, 2.20; 95% CI, 1.47–3.31; *P*<0.001). When stratified by the types of glaucoma, combination of *GSTM1* and *GSTT1* null genotypes was associated with increased risk of POAG (OR, 1.90; 95% CI, 1.15–3.13; *P* = 0.013) but not other types of glaucoma (OR, 3.04; 95% CI, 0.95–9.72; *P* = 0.061). We also examined if the risk of glaucoma was associated with combinations of *GSTP1* and *GSTM1*, or *GSTT1* genotypes, in which the homozygous Ile/Ile genotype for *GSTP1* was used as reference and the individuals heterozygous and homozygous for the Ile 105 Val allele was combined [22]. The results showed that that increased glaucoma risk was associated with the combined *GSTM1* null and *GSTP1* Val genotypes (OR, 1.86; 95% CI, 1.15–3.01; *P* = 0.012), but the combined *GSTT1* null and *GSTP1* Val genotypes played a protective role in glaucoma risk which, which remained of borderline statistical significance (OR, 0.60; 95% CI, 0.36–1.00; *P* = 0.051).

**Table 4:** Subgroup Analysis of the Association between *GSTM1*, *GSTT1* and *GSTP1* Polymorphisms and the Risk for Glaucoma.

Groups*	n†	StatisticalMethod	OR (95% CI)	*P*
***GSTM1 GSTT1***				
Pooled				
*GSTM1* null	7	Random	1.42 (0.66- 3.04)	0.373
*GSTT1* null	7	Random	1.79 (0.76- 4.21)	0.180
*GSTM1* null+*GSTT1* null	7	Fixed	2.20 (1.47- 3.31)	<0.001
POAG				
*GSTM1* null	5	Random	1.32 (0.43- 4.08)	0.633
*GSTT1* null	5	Random	1.99 (0.64- 6.19)	0.236
*GSTM1* null+*GSTT1* null	5	Fixed	1.90 (1.15- 3.13)	0.013
Others*				
*GSTM1* null	3	Random	2.72 (1.05- 7.00)	0.039
*GSTT1* null	3	Random	2.40 (0.61- 9.53)	0.212
*GSTM1* null+*GSTT1* null	3	Random	3.04 (0.95- 9.72)	0.061
***GSTM1 GSTP1***				
Pooled				
*GSTM1* null	3	Fixed	1.16 (0.74- 1.83)	0.523
*GSTP1* Val allele	3	Fixed	0.69 (0.44- 1.07)	0.099
*GSTM1* null+*GSTP1*Val allele	3	Fixed	1.86 (1.15- 3.00)	0.011
***GSTT1 GSTP1***				
Pooled				
*GSTT1* null	3	Fixed	1.07 (0.62- 1.83)	0.818
*GSTP1* Val allele	3	Fixed	1.17 (0.81- 1.69)	0.395
*GSTT1* null+*GSTP1*Val allele	3	Fixed	0.60 (0.36- 1.00)	0.051

* Others: including exfoliative, pseudoexfoliative and primary closed angle glaucoma.

† n: number of studies.

Abbreviations: POAG, primary closed angle glaucoma.

### Potential Publication Bias and Sensitivity Analysis

**Figure 2:**
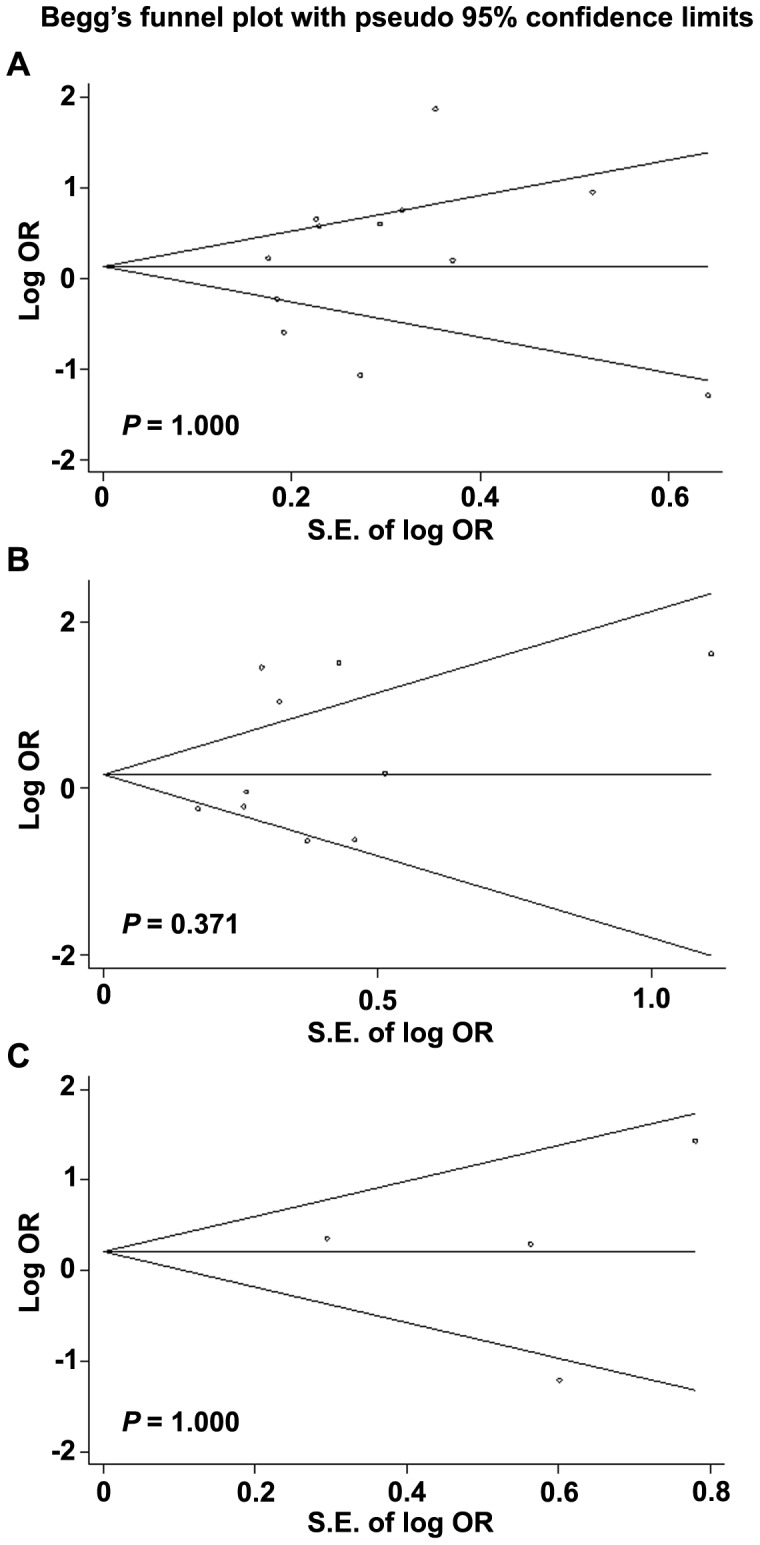
Funnel plots showed symmetric distribution. Log OR is plotted against the standard error of log OR for studies on *GSTM1* null (A), *GSTT1* null (B) and *GSTP1* Ile 105 Val (recessive model) (C) polymorphism. The dots represent specific studies for the indicated association.

Publication bias was firstly detected by Begg’s test for the overall pooled analysis of *GSTM1* and *GSTT1* null genotypes, and the recessive model of *GSTP1* polymorphism. The Begg’s test showed that the *P* value for *GSTM1*, *GSTT1* and *GSTP1* polymorphism was 1.00, 0.371 and 1.00 respectively, and the corresponding funnel plots showed symmetric distribution (Figure 2). The Egger’s test also showed that All the *P* values were more than 0.05 (Data not shown). Thus, no evident publication bias was found in present study. Sensitivity analysis was conducted by deleting each study in turn from the pooled analysis to examine the influence of the removed data set to the overall ORs. As shown in Figure S4, S5, S6, exclusion of each study did not influence the result in specific genotype comparison for *GST* polymorphism, suggesting that the results of synthetic analysis were robust.

## Discussion

In the present study, we systemically reviewed all available published studies and performed a meta-analysis to examine the association between the *GST* polymorphisms and susceptibility to glaucoma. Our meta-analysis showed that single *GSTM1* or *GSTT1* null polymorphism was not associated with glaucoma risk, and *GSTP1* Ile 105 Val polymorphism in recessive model was positively correlated with increased glaucoma risk. The combination of *GSTM1* null and *GSTT1* null, or *GSTM1* null and *GSTP1* genotype was associated with increased risk of glaucoma. Although different types of glaucoma have their own clinical characteristics and pathogenesis, our meta-analysis indicates that *GST* polymorphisms may contribute to increased risk of glaucoma.

Previously, the study by Juronen et al. suggests that the *GSTM1* positive phenotype may be a genetic risk factor for development of POAG [20]. However, the following studies show that the *GSTM1* null genotype is a risk factor for development of POAG [22], [26], while another study showed otherwise [33]. By pooled 10 studies, we did not find an association between *GSTM1* polymorphism and POAG, suggesting that previous controversial data may be due to small size of population. As to *GSTP1*, previous studies did not identify differences between POAG patients and control individuals in the frequencies of *GSTP1* Ile 105 Val genotypes [20], [22], [26], [28]; however, by pooled these studies, we found that *GSTP1* polymorphism was significantly correlated with increased POAG risk in Caucasian in a recessive model. It should be noted that the statistical significance of the association between the *GSTP1* polymorphism and POAG risk was at borderline level. The *GSTP1* 105-Val allele homozygote was correlated with increased POAG risk but the difference did not reach statistical significance (OR, 1.55; 95% CI, 0.94–2.55). Since the studies included were very limited, it is necessary to validate the association between *GSTP1* Ile 105 Val polymorphism and glaucoma risk in future studies.

To the best of our knowledge, this is the first meta-analysis assessing the association between single *GST* polymorphism, or combination of *GST* polymorphisms and glaucoma. Previously, Yildirin et al. reported an increasing glaucoma risk with higher numbers of the combined of *GSTM1* null and *GSTP1* 105-Val allele genotypes but this association was not significant [22]. Our meta-analysis results showed that the association between combined of *GSTM1* and *GSTT1* null genotypes, or *GSTM1* null and *GSTP1* Val genotypes, and the risk for glaucoma is statistically significant in Caucasians. The study by Yildirin et al. also found a trend of increasing glaucoma risk with higher numbers of the combined *GSTM1* null, *GSTT1* null and *GSTP1* 105-Val allele genotypes (OR, 2.3; 95% CI: 0.75–7.08) [22]. Due to the limited studies, we did not perform meta-analysis for association between the combined *GSTM1* null, *GSTT1* null and *GSTP1* 105-Val allele genotypes and glaucoma risk. The current available data support the multifactorial nature of glaucoma, and both genetic and environmental factors are involved in pathogenesis of glaucoma. However, most studies did not provide *GST* polymorphisms when stratified by environmental factors (e. g., smoking). The relationship between polymorphic *GST* with other genetic and environmental glaucoma risk factors may be highly complicated, and extensive research is required to ascertain how exactly the *GST* genotype affects the individual susceptibility to glaucoma.

Meta-analysis has advantages compared to individual studies, however, some potential limitations in our study should be considered. First, this meta-analysis was limited by the small sample size, especially in subgroup analysis aforementioned (e.g., studies on *GSTP1* polymorphism), which needs further investigations. Second, basic methodological differences among the studies might have affected the results. In addition to three studies [20], [22], [28], the controls recruited in other studies were hospital-based [21], [23], [24], [25], [26], [27], [29], [32], [33]. Most of studies used PCR methods for genotyping, but the study by Juronen et al. [20] used enzyme-linked immunosorbent assay. Although excluding this study did not affect the result of *GSTM1* and *GSTT1* genotypes, the association between *GSTP1* polymorphism and POAG risk in Caucasian was not significant due to the limited studies (n = 2) (Data not shown). Third, the studies differed in their procedure for sampling. Three studies used trabeculectomy specimens [25], [32], [33] while other studies used blood. We found no association between each single *GST* polymorphism and glaucoma risk in each subgroup analysis when the samples were stratified as trabeculectomy specimens or blood, and excluding one study using trabeculectomy specimens as samples did not affect the results on the associations between the combinations of *GST* polymorphisms and glaucoma risk (Data not shown). Fourth, most of the studies included in this meta-analysis did not categorize the POAG patients as high- and normal-tension glaucoma. So, we did not analyze the association between *GST* polymorphism and risk of high- or normal-tension glaucoma, and future studies should address this point. Last, the Caucasian group might have been genetically heterogeneous, with differences in terms of lifestyle and environment (e.g., European *vs*. Arabian). These factors may explain the heterogeneity in meta-analysis for Caucasian populations.

In summary, the present meta-analysis suggested that combination of *GSTM1* and *GSTT1* null genotypes, and *GSTM1* null and *GSTP1* 105-Val allele genotypes are associated with increased risk for glaucoma in Caucasian populations. The association between single *GST* polymorphism and glaucoma is either negative or evidence limited. More epidemiologic studies are suggested to further ascertain the relationship between *GST* polymorphisms and genetic predisposition to glaucoma.

## Supporting Information

Figure S1 **Forest plots of the association between** ***GSTM1*** **null polymorphism and glaucoma risk.**
(DOC)

Figure S2 **Forest plots of the association between** ***GSTT1*** **null polymorphism and glaucoma risk.**
(DOC)

Figure S3 **Forest plots of the association between** ***GSTP1*** **Ile 105 Val polymorphism and glaucoma risk.**
(DOC)

Figure S4 **Sensitivity analysis for** ***GSTM1*** **null polymorphism.**
(DOC)

Figure S5 **Sensitivity analysis for** ***GSTT1*** **null polymorphism.**
(DOC)

Figure S6 **Sensitivity analysis for** ***GSTP1*** **Ile 105 Val polymorphism.**
(DOC)

Table S1 ***GSTM1 and GSTT1*** **polymorphism genotype distribution of each study included in the meta-analysis.**
(DOC)

Table S2 ***GSTP1*** **Ile 105 Val polymorphism genotype distribution of each study included in the meta-analysis.**
(DOC)
