# Supplementary material for: Association of Glutathione S transferases Polymorphisms with Glaucoma: A Meta-Analysis
Source: PLoS One. 2013 Jan 14;8(1):e54037. doi: 10.1371/journal.pone.0054037 (PMC3544666; doi:10.1371/journal.pone.0054037)
Supplement: Figure S1 — Forest plots of the association between GSTM1 null polymorphism and glaucoma risk. (DOC) [file pone.0054037.s001.doc]

**Supporting Information Figure S1**


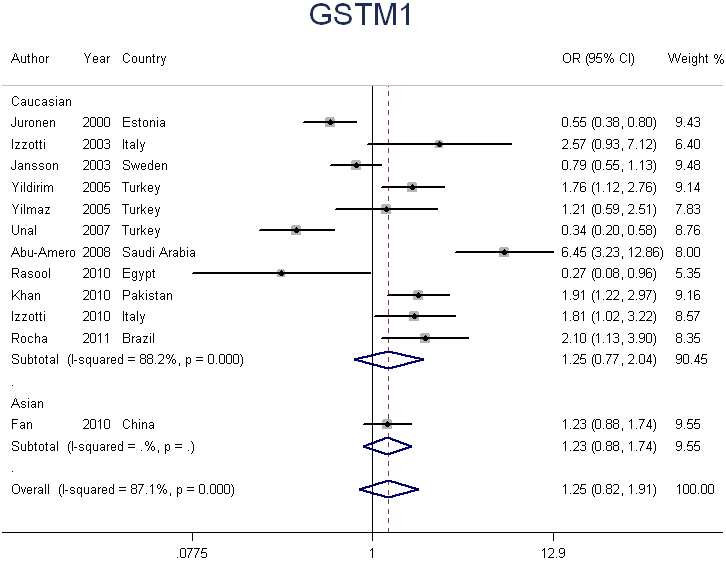


**Figure S1 Forest plots of the association between *GSTM1* null polymorphism and glaucoma risk.** The random-effects model was used to calculate the pooled effect estimates. The squares and horizontal lines correspond to OR and 95% CI of specific study, and the area of squares reflects study weight (inverse of the variance). The diamond represents the pooled OR and its 95% CI.
