# Supplementary material for: Association of Glutathione S transferases Polymorphisms with Glaucoma: A Meta-Analysis
Source: PLoS One. 2013 Jan 14;8(1):e54037. doi: 10.1371/journal.pone.0054037 (PMC3544666; doi:10.1371/journal.pone.0054037)
Supplement: Figure S3 — Forest plots of the association between GSTP1 Ile 105 Val polymorphism and glaucoma risk. (DOC) [file pone.0054037.s003.doc]

**Supporting Information Figure S3**


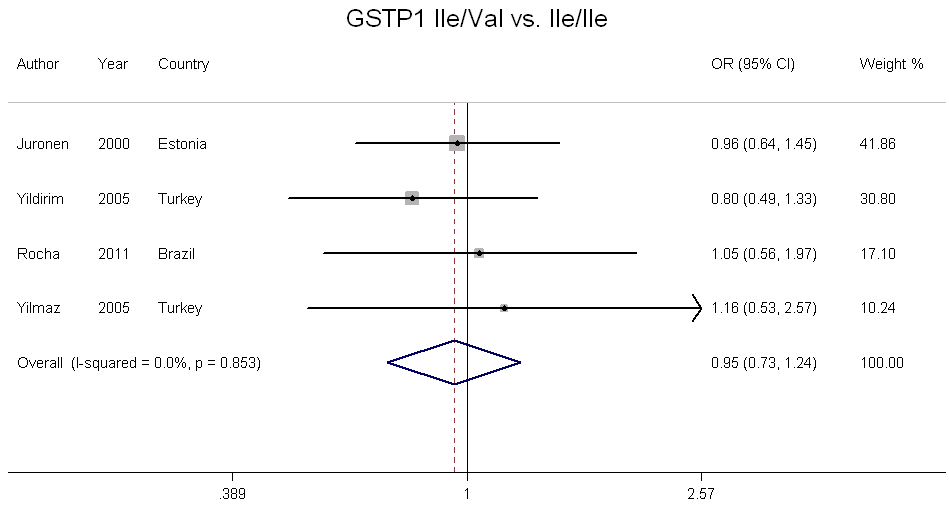


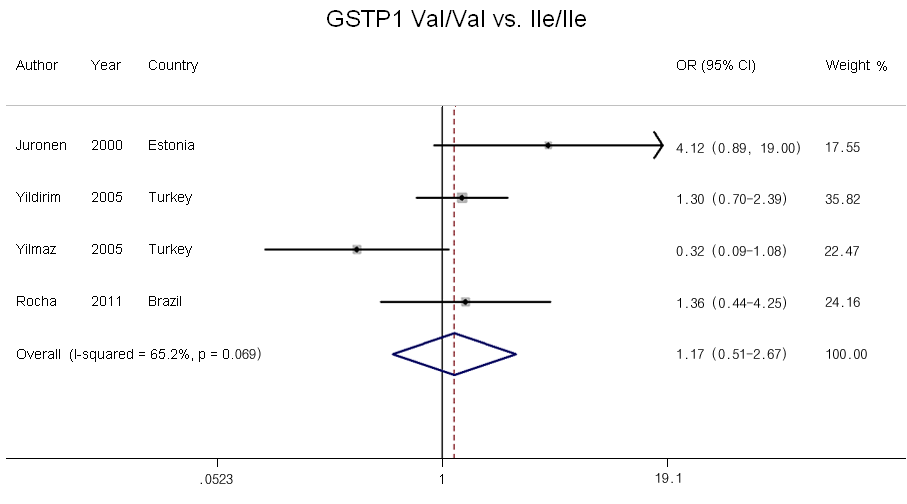


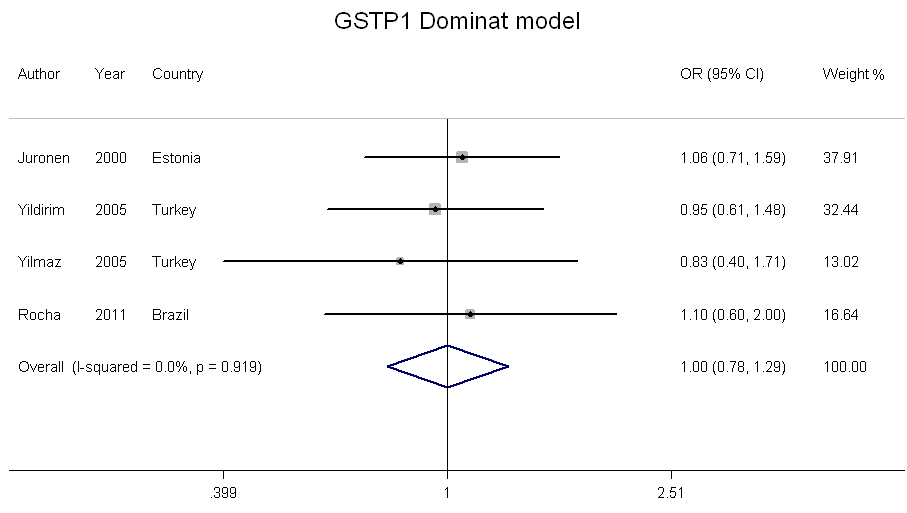


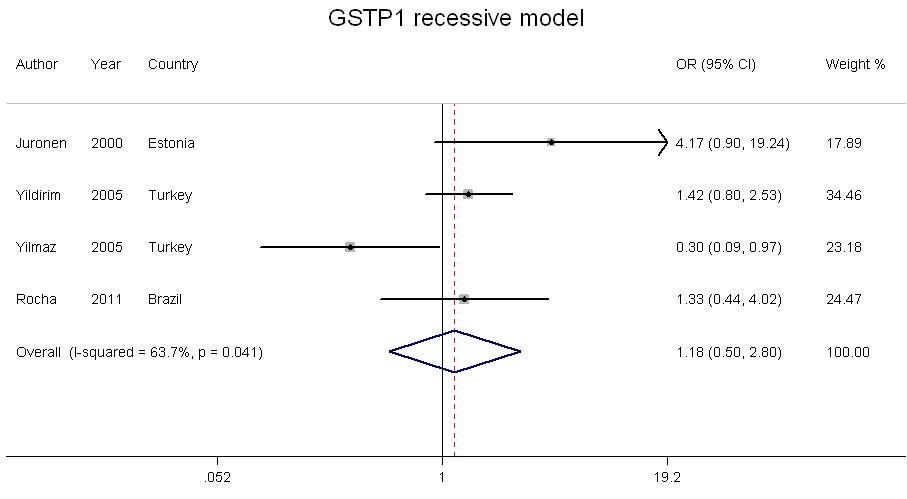


**Figure S3 Forest plots of the association between *GSTP1* Ile 105 Val polymorphism and glaucoma risk.** The random-effects (for homozygote and recessive) or fixed-effects (for heterozygote and dominant) model was used to calculate the pooled effect estimates. The squares and horizontal lines correspond to OR and 95% CI of specific study, and the area of squares reflects study weight (inverse of the variance). The diamond represents the pooled OR and its 95% CI.
