# Supplementary material for: Association of Glutathione S transferases Polymorphisms with Glaucoma: A Meta-Analysis
Source: PLoS One. 2013 Jan 14;8(1):e54037. doi: 10.1371/journal.pone.0054037 (PMC3544666; doi:10.1371/journal.pone.0054037)
Supplement: Figure S4 — Sensitivity analysis for GSTM1 null polymorphism. (DOC) [file pone.0054037.s004.doc]

**Supporting Information Figure S4**


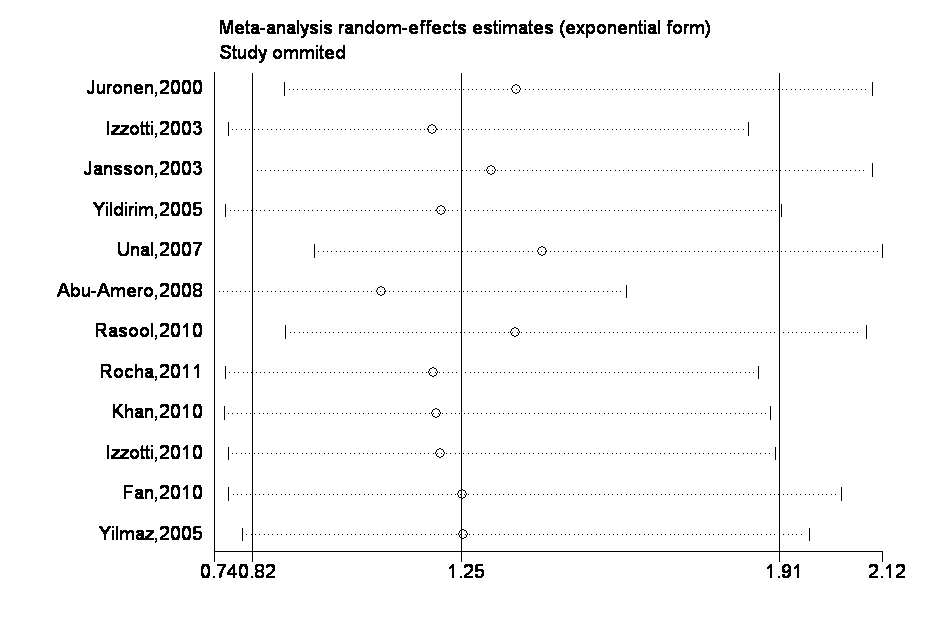


**Figure S4 Sensitivity analysis for *GSTM1* null polymorphism.** Each study was deleted at a time in synthetic analysis to detect the influence of the omitted study. The hollow circles represent OR of pooled results with the deletion of each study. The ranges of horizontal dotted-lines represent the 95% confidence intervals of the corresponding OR.
