# Supplementary material for: Association of Glutathione S transferases Polymorphisms with Glaucoma: A Meta-Analysis
Source: PLoS One. 2013 Jan 14;8(1):e54037. doi: 10.1371/journal.pone.0054037 (PMC3544666; doi:10.1371/journal.pone.0054037)
Supplement: Figure S6 — Sensitivity analysis for GSTP1 Ile 105 Val polymorphism. (DOC) [file pone.0054037.s006.doc]

**Supporting Information Figure S6**


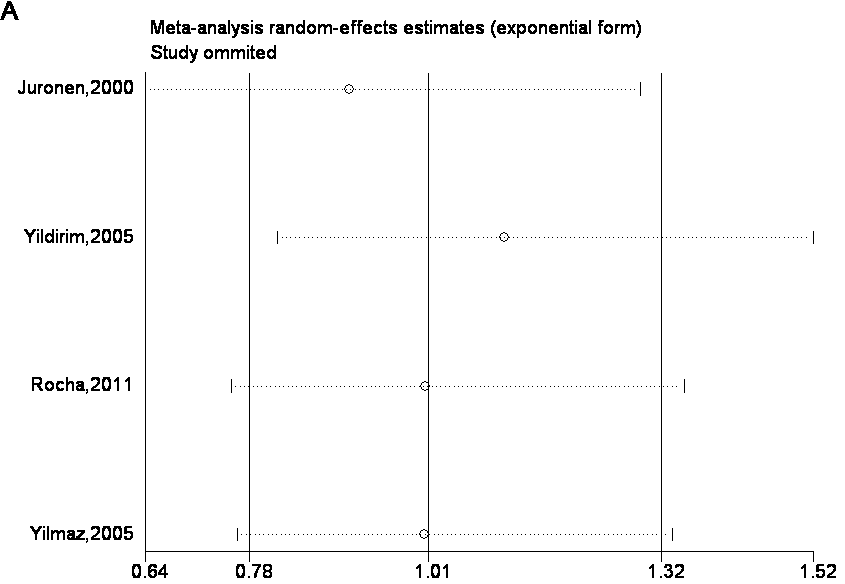


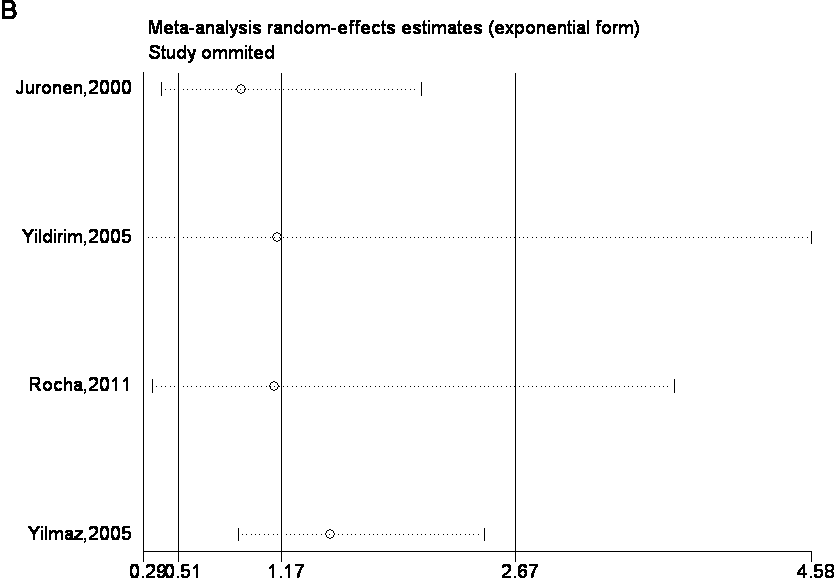

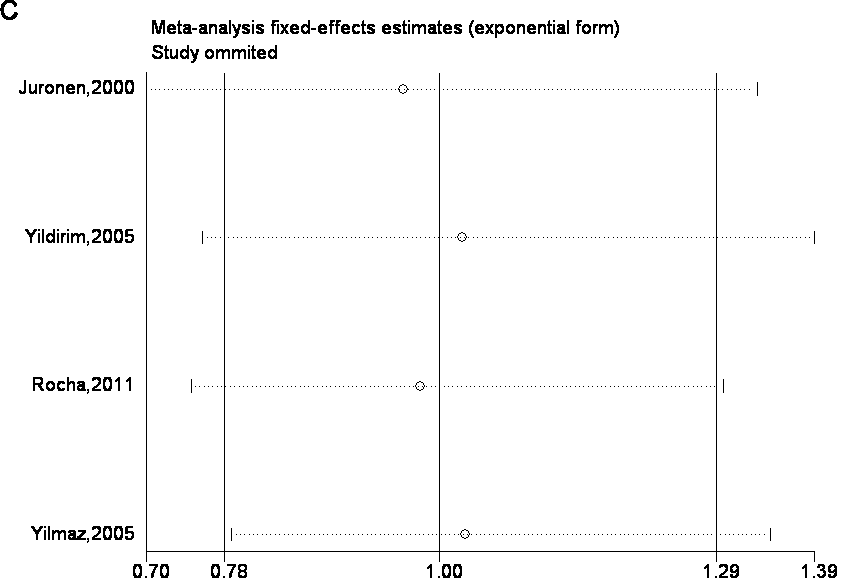


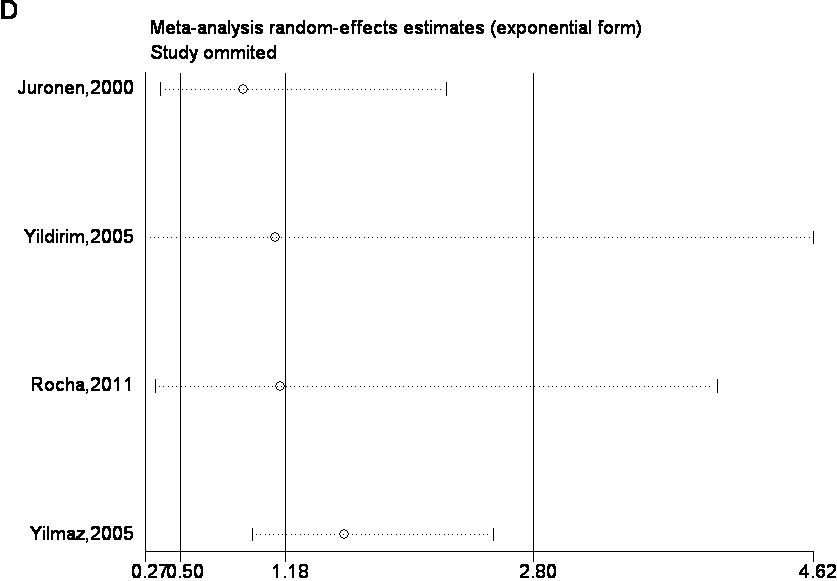


**Figure S6 Sensitivity analysis for *GSTP1* Ile 105 Val polymorphism.** Sensitivity analysis for *GSTP1* Ile 105 Val polymorphism in heterozygote (A), homozygote (B), dominant (C) and recessive model (D). Each study was deleted at a time in synthetic analysis to detect the influence of the omitted study. The hollow circles represent OR of pooled results with the deletion of each study. The ranges of horizontal dotted-lines represent the 95% confidence intervals of the corresponding OR.
