# Supplementary material for: Association of Glutathione S transferases Polymorphisms with Glaucoma: A Meta-Analysis
Source: PLoS One. 2013 Jan 14;8(1):e54037. doi: 10.1371/journal.pone.0054037 (PMC3544666; doi:10.1371/journal.pone.0054037)
Supplement: Table S1 — GSTM1 and GSTT1 polymorphism genotype distribution of each study included in the meta-analysis. (DOC) [file pone.0054037.s007.doc]

**Table S1 *GSTM1 and GSTT1* polymorphism genotype distribution of each study included in the meta-analysis**

| Author/ Year | Case | | Control | |
| --- | --- | --- | --- | --- |
|  | Wild-type | Null | Wild-type | Null |
| *GSTM1* |  |  |  |  |
| Juronen 2000 [17] | 150 | 100 | 91 | 111 |
| Izzotti 2003 [18] | 7 | 28 | 18 | 28 |
| Jansson 2003 [30] | 171 | 217 | 69 | 111 |
| Yilmaz 2005 [25] | 26 | 27 | 35 | 30 |
| Yildirim 2005 [19] | 69 | 84 | 94 | 65 |
| Unal 2007 [20] | 113 | 31 | 67 | 54 |
| Abu-Amero 2008 [21] | 60 | 47 | 107 | 13 |
| Rasool 2010 [22] | 22 | 10 | 6 | 10 |
| Fan 2010 [26] | 221 | 184 | 120 | 81 |
| Khan 2010 [24] | 80 | 85 | 104 | 58 |
| Izzotti 2010 [29] | 32 | 68 | 46 | 54 |
| Rocha 2011 [23] | 44 | 43 | 58 | 27 |
| *GSTT1* |  |  |  |  |
| Juronen 2000 [17] | 213 | 37 | 166 | 36 |
| Izzotti 2003 [18] | 10 | 13 | 22 | 24 |
| Yilmaz 2005 [25] | 44 | 9 | 47 | 18 |
| Yildirim 2005 [19] | 115 | 38 | 118 | 41 |
| Unal 2007 [20] | 74 | 70 | 99 | 22 |
| Abu-Amero 2008 [21] | 81 | 26 | 112 | 8 |
| Rasool 2010 [22] | 24 | 8 | 15 | 1 |
| Fan 2010 [26] | 217 | 188 | 95 | 106 |
| Khan 2010 [24] | 126 | 39 | 146 | 16 |
| Rocha 2011 [23] | 72 | 15 | 61 | 24 |
