# Supplementary material for: Association of Glutathione S transferases Polymorphisms with Glaucoma: A Meta-Analysis
Source: PLoS One. 2013 Jan 14;8(1):e54037. doi: 10.1371/journal.pone.0054037 (PMC3544666; doi:10.1371/journal.pone.0054037)
Supplement: Table S2 — GSTP1 Ile 105 Val polymorphism genotype distribution of each study included in the meta-analysis. (DOC) [file pone.0054037.s008.doc]

**Table S2 *GSTP1* Ile 105 Val polymorphism genotype distribution of each study included in the meta-analysis**

| Author/ Year | Case | | | Control | | | |
| --- | --- | --- | --- | --- | --- | --- | --- |
|  | Ile/Ile | Ile/Val | Val/Val | Ile/Val | | Val/Val | Ile/Val |
| Juronen 2000 [17] | 170 | 70 | 10 | | 140 | 60 | 2 |
| Yildirim 2005 [19] | 75 | 46 | 32 | | 76 | 58 | 25 |
| Yilmaz 2005 [25] | 27 | 22 | 4 | | 30 | 21 | 14 |
| Rocha 2010 [23] | 44 | 35 | 8 | | 45 | 34 | 6 |
